# Comparison of social resistance to Ebola response in Sierra Leone and Guinea suggests explanations lie in political configurations not culture

**DOI:** 10.1080/09581596.2016.1252034

**Published:** 2016-11-09

**Authors:** Annie Wilkinson, James Fairhead

**Affiliations:** ^a^ Institute of Development Studies, University of Sussex, Brighton, UK; ^b^ Department of Anthropology, University of Sussex, Brighton, UK

**Keywords:** Ebola, resistance, trust

## Abstract

Sierra Leone and Guinea share broadly similar cultural worlds, straddling the societies of the Upper Guinea Coast with Islamic West Africa. There was, however, a notable difference in their reactions to the Ebola epidemic. As the epidemic spread in Guinea, acts of violent or everyday resistance to outbreak control measures repeatedly followed, undermining public health attempts to contain the crisis. In Sierra Leone, defiant resistance was rarer. Instead of looking to ‘culture’ to explain patterns of social resistance (as was common in the media and in the discourse of responding public health authorities) a comparison between Sierra Leone and Guinea suggests that explanations lie in divergent political practice and lived experiences of the state. In particular the structures of state authority through which the national epidemic response were organised integrated very differently with trusted institutions in each country. Predicting and addressing social responses to epidemic control measures should assess such political-trust configurations when planning interventions.

## Introduction

The West African Ebola epidemic which begun in December 2013 has far eclipsed all other Ebola outbreaks put together with official figures counting 28,637 cases, as of December 2015 (World Health Organisation [WHO], 2015a), with ongoing flare-ups since the control of the initial outbreak (Centres for Disease Control and Prevention [CDC], 2016). The three Mano River countries of Guinea, Sierra Leone and Liberia were hardest hit. This paper examines the roots of social resistance to Ebola control measures. Such resistance was marked in all three countries and without it the epidemic would have been over sooner (WHO, 2015b). Of interest here are the differences in the pattern and intensity of resistance which we explore by comparing Guinea and Sierra Leone.

Whilst many people across the three countries welcomed epidemic control measures and sought assistance, responders also experienced resistance, ranging from the passive to violent conflict and overt rejection of control stipulations. Guinea saw continuing episodes of violence and outright resistance. As well as the murders of eight outreach workers in Womey in September 2014, and of two policemen in January 2015, WHO situation reports from mid-2014 to mid-2015 document challenges in community engagement, regular clashes, and the spurning of help (see also ACAPS, 2015).^1^ In Sierra Leone reactions initially included denial, riots and the stoning of vehicles. However, by the end of 2014 social responses to Ebola control had mostly shifted from these flashpoints to an apparent acceptance of extraordinary interventions. Why was Sierra Leone, after 10 years of brutal civil conflict, more able to settle into Ebola management when parts of Guinea were not?

To address this question, one first needs to ask if and when ‘resistance’ is actually resistance. Actions that are read as resistance are often nothing of the sort. Alternative reasons the ill might not present at Ebola Treatment Units (ETUs) include the financial and logistical impossibility of getting to them; the absence of safe transport; a lack of child-care; or a lack of the social and economic capital needed to survive quarantine. Resistance cannot be inferred from ‘non-compliance’. In Guinea, authorities adopted the term ‘reticence’ to distinguish reluctance from oppositional resistance (ACAPS, 2015). For response workers, reticence could be inferred from unreported or absconded cases, or so-called ‘swab positives’ (i.e. those who are only diagnosed from results of swabs taken upon death). This apparent reluctance may well have been due to the above concerns. The focus here is with more clearly ‘intentional’ acts of resistance, which were more common in Guinea (ACAPS, 2015).

Our comparison of Guinea and Sierra Leone is based on press and technical reports of the epidemic in each country, and personal involvement in the response as members of the Ebola Anthropology Response Platform.^2^ In this capacity we had regular contact with public health officials and (AW) made three trips to Sierra Leone during the epidemic. To explain the different trajectories of social responses to control measures we reviewed historical, political and anthropological literature on each country, and draw on our own longstanding research engagement in the region. We begin by first establishing some ways in which Guinea and Sierra Leone are similar in order to discount simple cultural explanations for observed differences. Then follows a section each on Guinea and Sierra Leone where we consider how different styles of statecraft appear to have shaped the logics and intensity of resistance and the availability of local-level institutions for national and international responses to work through. Finally we discuss some alternative explanations for national differences and consider the implications of our argument for dealing with resistance in public health.

Sierra Leone and Guinea are two of the poorest countries in the world, consistently hovering around the bottom of health and development indices. International Monetary Fund lending policies have required that public spending on health care is limited (Kentikelenis, King, McKee, & Stuckler, 2015), and aside from a few vertical programmes, the majority of people in each country have to pay for services from a mix of public and private suppliers (Bloom, MacGregor, McKenzie, & Sokpo, 2015; Leach, Fairhead, & Millimouno, 2008). The public system is severely understaffed in each country and both face challenges in the training and retention of health providers. These conditions are a large part of what made people reluctant or unable to visit medical facilities early in the epidemic. Yet the same problems were pervasive in each country. Both countries have also had experiences with other epidemic diseases including Lassa fever (Mylne et al., 2015), sleeping sickness (Simarro, Diarra, Ruiz Postigo, Franco, & Jannin, 2011) and smallpox (Rashid, 2012).

The afflicted regions of the Mano River states have many social similarities linked to historic interchange and creolisation. Although people are familiar with Western health care and make use of biomedical products, it is alongside existing understandings of disease and ways of managing health that share much in common (Højbjerg, 2007; Jambai & MacCormack, 1996; McGovern, 2012). Ill health might be relieved by biomedicine but the causes and treatment often require a different sort of attention – to social faults, the anger of the deceased or of other spirits, or the maleficent work of sorcerers. Sudden sickness and death – as with Ebola – is often associated with un-natural occurrences, normally understood to follow either a ‘social fault’ or a curse. Reparation of the socio-ecological order, confessions and other forms of protection are often required to correct the causal transgressions or to counter aggressions. Interfering with these practices can generate friction with local political institutions as rites are overseen by specialists that in many parts of the region include officials of the gendered initiation societies, or religious leaders whether Imams or Pastors. Political leaders may have secular power but society officials and religious leaders often have ‘real’ control, the former managing the socio-ecological processes which link land ownership, farming practices, resource management, health, fertility and death (Fairhead & Leach, 1996).

Much ‘resistance’ stemmed from the way Ebola control operated with little consideration for these sensitivities (Anoko, 2014; Fairhead, 2016). Important to this analysis, however, is that these more ‘cultural’ tensions are common to the social worlds on both sides of the Guinea/Sierra Leone border. To explain the contrast in acts of defiant resistance, it is necessary to ask how these social and cultural orders intersect with the political and administrative structures.

Again there are similarities. The ambivalent view Sierra Leoneans and Guineans (and Liberians) have of their states and of ‘white people’ stem from past and present experiences of elites and outsiders extracting both human and natural resources at the expense of local populations. These experiences, from slavery to diamonds and other minerals, have created logics of distrust and secrecy (Ferme, 2001; Shaw, 2002). Corruption scandals, such as over the handling of licenses to mine for iron ore at Simandou in Guinea (Kochan, 2013), or the misuse of donor funds intended for health system strengthening programmes in Sierra Leone (GAVI Vaccine Alliance, 2013) – to name just two – affirm this logic of underhand and ill-gotten gain at the expense of African minerals and bodies.

Any new pole of resistance is inevitably entangled with existing social divisions, whether concerning ethnicity, party-politics and so on. It is here that we now pick up on the contrasting ways that Ebola and responses to it have played into ethnic and party-political relations in the region. It is the practices of state formation and administration that have been so different in Sierra Leone and Guinea.

## Guinea

The Ebola spillover occurred near Guéckédou in a Kissi-speaking part of the Forest Region of Guinea; a region with a political history of marginality, trading cola nuts with the precolonial Manding savanna empire of the thirteenth and sixteenth centuries, but also suffering periodic conquest and slave raids (Fairhead, Geysbeek, Holsoe, & Leach, 2003).

When this region of Forest Guinea was incorporated into Euro-American trade networks from the fifteenth century, it was initially as an exporter of the iron smelted locally, but it was then also plundered for Atlantic slavery before being subjugated violently by European colonies in the nineteenth century. Crucially, Atlantic slavery and colonisation were experienced in this region not just as an encounter with the Euro-American world, but with Manding warlords who profited from the slavery and then the rivalry between colonial powers. This heightened antagonism between inhabitants of the Forest Region and both the colonial (Christian) world and the Manding and increasingly Muslim political orders entangled with it (Anoko, 2014; Højbjerg, 2007; Iffono, 2010; McGovern, 2012).

In these periods, the initiation societies that coordinate the cycle of life and prosperity in Forest Guinea also orchestrated political defence, solidarity and confederacy, and drilled their initiates in the military arts of protection, concealment, defence and battle. They regulated trade routes, markets and monetary value.

Subsequent history has kept alive these social and political cleavages. The Independent Marxist revolutionary movement that won Guinea’s Independence followed an aggressive policy of ‘demystification’ against initiation societies, destroying their ritual objects, exposing their secrets, and banning ceremonies in an attempt to change mentalities in the pursuit of national unity, modernity and African communism (McGovern, 2012). This was experienced as political subordination of the region to the Manding and Islamic north (e.g. the Malinke, Konyanke & Toma-Mania) as the revolutionary President, Sekou Touré was understood as ‘from there’. Revolutionary land reform was experienced in the forest region as a threat to indigenous land rights and as favouritism to rival Manding immigrants (Højbjerg, 2007; McGovern, 2012). Although initiation practices were banned, their healing and burial practices continued in secrecy, helped by their legality across the land borders in Sierra Leone and Liberia. The effect of this suppression from 1958 to 1984 was to further politicise and objectify this order of things as a ‘religion’ that unified its people, so that from the 1990s, when restrictions on initiation societies were lifted, all politicians from the Forest Region were obliged to turn to initiation societies to rally political support. Even the urban educated ‘Christianised’ elite of the Forest Region nurtured their political aspirations by supporting them (Højbjerg, 2007; McGovern, 2012).

This ‘revivalism’ was thus entirely entwined with national politics, in which initiation society activities have been drawn on to produce not only a pan-Kissi, and a pan-Loma political identity (as well as Kpélé, Guerzé, Kono & Mano ones), but due to their common historical experiences, a pan-Forest Region identity which enabled the political leaders of each language area to ally and form a power bloc in national politics.

Forest Region initiation societies have remained central to political action and prosperity. Neither Islam nor the Christian missions easily found local converts. In 1989, religious surveys in Macenta indicated that only 7% of Loma people professed Christian faith, whereas 78% followed traditional practices (Højbjerg, 2007, pp. 64, 65). The 15% professing as Muslims were ethnic rivals living in the region.

The Forest Region has continued to be subordinated to other regions in subsequent national politics and its politicians and inhabitants have become highly sensitive to a ‘conspiracy of power’ that favours immigrant rivals and the white world, giving them control over indigenous resources, whether land or its globally significant iron ore, gold and diamond deposits. Their initiation societies remilitarised during the turbulent years of the Liberian and Sierra Leonean civil wars and became the locus of civil defence, with foes again being those of Manding/Islamic warring factions. Since then, episodes of sectarian violence especially between Loma and Manding have heightened tensions between those of the forest region (*Forestiers*) and those they would see as ‘immigrants’ (Guinéenews, 2014a, 2014b).

A military coup in 2008 did install a Forestier, Moussa Dadis Camara, as President. He is Guerze (from Lola Prefecture) and drew on initiation societies to build ethnic militias. His political ascendency was short lived, however, as well as murderous and is perceived locally to have been opposed by the ‘white’ international community. He was deposed in 2009 after an assassination attempt.

The social and religious practices of the *Forestiers* are disdained by many in the rest of Guinea that is Islamic, by the white Christian world and by the modernity of revolutionary African nationalists. As Ebola initially spread in the forest region, inhabitants elsewhere cast it as an ethnic disease (a disease of Kissi or of *Forestiers* (Camara & Lazuta, 2015). The disease was thus quickly stigmatised within national ethnic stereotypes. The moral ‘well’ did not envisage getting it and did not take well to being educated to avoid it – of being cast as immoral. One effect of this stigma was that good Muslims, and especially Imams and Muslim healers – who were at high risk of exposure given their role in healing and funerals for their community – were not quick to admit Ebola infection to themselves or others. In Macenta, Imams continued to wash the bodies of those who died for several months (until October 2014) and several were infected and died (La Croix, 2014). It was an Imam who travelled to Mali, avoiding road checkpoints, who introduced Ebola there in November 2014 (Penney & Farge, 2014). Imams led illegal funerals in the West of the country, the suppression of which caused riots in February 2015 (AFP, 2015).

Those from the Forest Region, too, resented being cast as immoral by Ebola. Many saw the phenomenon as a pretext for further suppression. Significantly, the revolutionary regime of Sekou Touré developed a powerful state apparatus in central government, and at the Prefecture and Sous-Prefecture levels, where government appointed employees represent the various ministries at every level. Building on French colonial practice, the political tradition developed that these civil servants would not usually be from the region where they were employed – in contrast with ‘traditional leaders’ be they elders, members of founding families, or descendants of colonial chiefs (Schroven, 2010). Since the Presidency of the Sekou Touré (1958–1984), but especially since 2009 under the current democratic Presidency of Alpha Condé, also from the Manding region, not only have *Forestiers* been governed by civil servants associated with the regions of their historic antagonists, but they assert, too, that they are discriminated against in recruitment for administrative appointments. As Guinéenews (2014a) asserted ‘This feeling of helplessness and impunity among aggressors has revived and exacerbated communal clashes’.

Sensitivities over stigma were multiplied when the ruling political elite, who are locally so distrusted, began to join Ebola ‘sensitisation’ missions and conduct public meetings in a style that confused them with electioneering. On 10 September, the four allied opposition parties from the Forest Region (that all draw on initiation societies for their powerbase) united to object to what they saw as this politicisation of Ebola sensitisation and called on public vigilance (Guinéenews, 2014b). Anxieties were raised when such a delegation arrived at the Sous-Prefecture of Womey. It included the Governor of the Forest Region and the Prefect of the Prefecture, who would speak only of Ebola and not the wider grievances that the community wanted to address to such leaders (Guinéenews, 2014a). A pastor of an evangelical church, who was intolerant of initiation societies and who worked for a US Christian relief agency, accompanied them (C M Alliance, 2014). This delegation knowingly coincided with the ‘coming out’ ceremony and festival for new girl initiates that attracted senior initiates from neighbouring villages. As the stench of bleach spray wafted over, inhabitants reasoned that the delegation was infecting the village, so the initiation societies orchestrated a ‘pre-emptive’ attack, killing those of the delegation they could and tracking down those who had escaped (Brittain, 2015; Ouendeno, in press). They then defended their village with ex-combatant machine guns until the Guinean military eventually intervened.

The politicisation of Ebola response also aggravated dissent elsewhere in Guinea, and in particular Forécariah Prefecture. This is a largely Muslim, Susu-speaking region and was politically favoured under the Presidency of Lansana Conte (1984–2008) and so aspects of the sensitivities in the Forest Region are not felt. Yet since 2008 when the locally supported Party of Unity and Progress party lost power, tensions have escalated between the state officials posted to the Prefecture, who are assumed as allied to the new ruling party (of Alpha Condé), and the local population. Youth had become more militant, organising, for example, to prevent an unpopular Prefect from speaking at high profile event. Ebola played into these tensions in September 2014 when a Red Cross team arrived to bury a prominent family matriarch, but were disrespectful when bagging the unwashed body and tossing it into a pick-up. At this, the family withdrew any support they had given, some youth reclaimed the body at knifepoint and another 3000 attacked the health centre, offices, vehicles and an ETU where WHO epidemiologists worked. The family proceeded to remove the body from the bag, wash and bury it (Africalog, 2014). Youth claimed to journalists that no Ebola protection kits had been distributed in the town, voicing that ‘Only some political parties would have helped the town’ – i.e. the locally supported party [probably the ‘Susu’ Union of Republican Forces] not the one in power (Guinéematin, 2014).

The political sensitivities were aggravated in this Forécariah region also by high level political delegations that conducted Ebola sensitisation. The Minister of Communication led a ‘high level delegation’ to Forécariah in December 2014 (UNMEER, 2014) despite being highly criticised by opposition leaders in October, for politicising Ebola sensitisation (Loudah, 2014). Soon after his visit, on 14 January 2015, when senior police arrived at the village of Laya and gave a healer medication, tragically he promptly died, and villagers accused the police of killing him and of spreading Ebola. They lynched two and seriously injured others (Diallo, 2015). At nearby Sinkine when health officials cleaned a water source, inhabitants noticed that they had killed frogs (Ebola messaging had heightened attention to the generalised death of bush animals) and assumed ‘that they came to kill us’ – despite it being the Prime Minister’s home village (Guinéematin, 2015). The widespread suspicion of the genocidal intent of the party in power made it impossible for Ebola response to operate in these locations, even though dozens were dying.

These examples show how non-compliance, fear and resistance are associated with a radical distrust of the party in power and those working for it. To quote one voice in a crowd, this was ‘Not a battle against the Red Cross, but against the [perceived] abuses of Mr. Condé’ (‘Abedi’ in Momou, 2015).

Interpreted in this way Ebola is not only playing into Guinea’s increasingly ethnicised politics, it is upping the stakes by transforming existing distrust and grievances into a fear of actual genocide. But how could people’s distrust in Guinea become as visceral as to think that Ebola was being introduced by outsiders purposefully ‘to destroy them’? The proximity of the most violent resistance to Simandou and Forécariah mining businesses – internationally recognised for corruption and locally known for their poor employment practices and unfulfilled promises to local communities – indicates the need also to examine how those in power are thought to enjoin with foreigners (‘whites’) to expropriate (steal) local resources. Sense can be made of the immoral, callous nature of this perceived robbery within narratives of malicious sorcery: ‘*le pouvoir*’ and the white world is more concerned with mining profit than people. These mines are the stuff of global businesses with white expatriate management clinching deals in Ministries and companies that have ‘never done anything for the village’ (Sylla, 2014) even though villages have traditional claims to the ore. Given this experience: ‘In some areas and in remote villages, people firmly believe in the version of the introduction of Ebola by whites who have mineral resource interests, with the complicity of the government for the sole purpose of destroying their communities’ (Guinéenews, 2014b; see also Fairhead, 2016).

## Sierra Leone

Ebola appeared first in the East of Sierra Leone, a region which, like its counterpart in Guinea, had long been integrated into West African trading routes, slavery and warfare, as well as rule under European colonialism. However a rather different blend of authority has emerged there.

Indigenous groups of Sierra Leone, including the Kissi, Limba, Temne, Kono, Sherbo and Koranko and Mende, have faced multiple waves of external influence. Beginning well before the sixteenth century, Mande traders, warriors and Islamic teachers advanced towards the coast from Manding territories North of present-day Sierra Leone (e.g. Mali) (Skinner, 1978). Traders with expansive networks became influential in local economies. Warriors either conquered and replaced existing leaders, or hired their services out to these ‘big men’. Through intermarriage these newcomers were integrated into existing farming settlements, which were organised around the lineage of original founders (hunters, warriors, pioneers) and those dependent on them or subjugated by them (Little, 1947; Skinner, 1978). Some were granted land rights and influence in indigenous political systems, watering down their ‘stranger’ status (Skinner, 1978). As well as bringing Islamic institutions with them they ushered in political systems based increasingly on personal prestige and military might, and societies engaged in petty warfare. Countering this civil power were the Poro (male) and Sande (female) initiation societies who regulated spiritual and socio-ecological orders and were understood as the ‘real’ custodians of the land and its people (Little, 1947).

On a different front, at the end of the fifteenth century Portuguese traders had first visited the Freetown peninsula, where the deep natural port meant that trade with Europeans flourished, especially in slaves. Local slaving economies and practices grew alongside the European one, which from the mid-seventeenth century crossed the Atlantic. Having outlawed the international slave trade in 1807, the British claimed Freetown as a Crown Colony in 1808. Domestic slavery, and associated raids and warfare continued outside the Colony, but beginning in 1787 the coast of Sierra Leone increasingly became home to colonies of freed slaves from London and North America. These colonisers established an antagonistic relationship with local leaders who reacted violently to the effective seizure of their land (Harris, 2014). Descended from these freed slaves, the Krios, speaking their own English-based creole, identified as superior to the ‘savage’ natives (Kandeh, 1992). But the Krio’s aspirations of superiority were tempered by their subjugation under British racism and colonial rule. Krio chances of hegemony were further squeezed by Lebanese traders and the gradual empowerment of indigenous populations, especially the largest Mende and Temne groups (Abraham, 1978; Harris, 2014).

The contemporary politics of Sierra Leone is shaped by the bifurcated system of governance set up by the British (Jackson, 2007). Though the Freetown area had become a Crown Colony in 1808, British interest in the land beyond it was initially limited. Indigenous rulers retained their sovereignty until 1896, when, to pre-empt French incursion via Guinea, the British established the Sierra Leonean Protectorate which covers what is now the rest of Sierra Leone. A system of native administration was designed which imposed a chieftaincy system in the Protectorate in order to collect taxes and monitor the population (Fanthorpe, 1998). The encroachment of the British and their insistence on a particularly heavy tax met with violent opposition – the ‘Hut Tax Wars’ of 1898 – across the North and East of the country. The insurrection was severely suppressed by the British (Rashid, 2011). After this defeat, the ‘laissez faire’ approach of arm’s length governance through the chiefdoms took root.

Although there had been famous rulers who controlled groupings of settlements there had been no central organisation and the colonial system of chiefs, elected for life, from ‘ruling houses’ was new (Fanthorpe, 1998; Little, 1947). Importantly, there was variation in the patterns and sources of authority of these pre-chiefdom rulers: some relied on ancestral claims to the land while others drew their strength from war, and varying degrees of assimilation into local lineages. When it came to identifying the first chiefs in the new colonial system this made claims to succession hard to verify. A gap was also left after the deaths or removal of leaders who had taken part in the Hut Tax Wars. Some people with tenuous claims were appointed as chiefs (Little, 1947).

Yet despite being something of an ‘invented tradition’, with some opportunistic entryists, chiefdom authority is at least in keeping with well-established patterns of ritual and pre-state political life which relies on claims to have roots in the land and to ancestors (Ferme, 2001; Murphy, 1980) or military domination (Little, 1947; Skinner, 1978). Local politics is, therefore, in the hands of ‘sons of the soil’ (or daughters), or those who trace their ancestry to highly regarded warriors. The result has been, at times, intense power struggles during chiefdom elections between those claiming ancestry to these houses (Fanthorpe, 1998; Ferme, 1998; Murphy, 1990). Chiefly legitimacy is further strengthened in some chiefdoms by an overlap between the society leaders and the chiefdom authorities (Fanthorpe, 2005), though in other areas where there is no overlap societies may be a check on chiefly power (Little, 1947).

In their mediating role between local populations and the colonial government chiefs sought to protect local populations and simultaneously to take advantage of their position. Chiefs colluded with their subjects to resist colonial attempts to control smallpox in 1905 and again in 1915 (Rashid, 2011). This included covering up cases and not enforcing anti-crowding measures. Yet they also routinely took cuts from resources which were flowing into chiefdoms. By and large this abuse was tolerated, with revolts directed elsewhere. For example, in 1919, severe food scarcity driven by bad harvests and shortage of labour due to conscription for the First World War, on the back of epidemics of smallpox and Spanish Flu, led to widespread riots. Yet anger was primarily directed at Lebanese traders who were accused of hoarding food and profiting from shortages (Rashid, 2011).

After independence the relationship between chiefs and national political authorities was consolidated as the patrimonial state took shape: chiefs depended on national politicians for resources to distribute among their networks while members of parliament depended on chiefs to secure votes in general elections (Jackson, 2005, 2007; Reno, 1995). Politicians took a less antagonistic approach towards indigenous spiritual-political orders than in Guinea. For example, as a young medical officer in the 1940s Milton Margai, who went on to become Sierra Leone’s first Prime Minister, had trained Sande officials in health and hygiene for them to incorporate safer practices into their initiation (circumcision) and delivery practices (Jambai & MacCormack, 1996).

Since independence from British rule, national politics has been dominated by two political parties: the Sierra Leone People’s Party (SLPP) and the All People’s Congress (APC). Each party draws on a regional and ethnic power base: the SLPP are supported in the East, a predominantly Mende area, and the APC are supported in the North by the Temne and also Limba. A 14-year period of one party rule and state decline, from 1971 to 1985, by Siaka Stevens of the APC preceded the country’s 11-year civil war.

When the first cases of Ebola were detected in Kenema and Kailahun in Eastern Sierra Leone, in late May 2014, the outlook was not promising. Ebola’s route into the country took the same path through Kailahun as the rebels had. As war memories stirred, lines were drawn down party-political and regional cleavages. Commentators pushed the war analogy to highlight the inadequacy of the government’s response in ways which recalled state failure, complacency and protracted violence: ‘When news of the Ebola virus in the country first broke in March this year, government took it with a pinch of salt, like the Joseph Saidu Momoh government took the Foday Sankoh war’ (Kamara, 2014). The accusation that the government failed to act decisively resonated because the current government – like that of Momoh – is APC. Early on, a common idea was that the epidemic was started – or was allowed to get out of control – by the Government in order to depopulate opposition areas (Kanu, 2014). Rumours abound that this was before a national census planned for December 2014. The collection of population data for tax and voting purposes has long been a fraught affair, with numbers often manipulated and tied to violence and injustice, or fear of it (Fanthorpe, 1998; Ferme, 1998). These undercurrents were fanned by partisan press. For example The New People, a pro-SLPP newspaper, reported sources saying ‘the Health Minister, Miatta Kargbo, is GUILTY of ordering the kidnap and possible secret killing of dying ebola virus patients’ (Fonti, 2014) in their reporting of one of a growing number of disturbances in Kenema and Kailahun during June and July where police used live rounds and tear gas to disperse protests. The Lassa ward at Kenema Government Hospital, where Ebola patients were initially taken, had historically been tainted by rumours of ‘lethal injections’ administered by the health care staff (Wilkinson, 2013).

In the first six months of the epidemic in Sierra Leone these tensions continued to build. In July 2014 there was a large riot in Kenema when crowds threatened to burn the hospital where the treatment centre was located down (Moriba, 2014). Accusations that the government was trying to wipe out opposition voters mixed with suggestions that it was a ploy by bankrupt Governments to get donor money or an opportunity to steal body parts. In addition to riots, families refused to allow their loved ones to be taken to Ebola wards (Forfana, 2014). From Kailahun (Mark, 2014) in the East, to Port Loko (Ismail, 2014) in the North West, ambulances and burial teams were stoned when they tried to collect patients and bodies.

However, reports of violence subsided and by November 2014 the Sierra Leonean Ebola response was being implemented without major or widespread incidents. This is startling as it involved some extraordinarily authoritarian interventions, enacted under State of Emergency regulations. Whole regions, villages and households have endured externally enforced quarantines. Quarantines were not widely used in Guinea. In both countries it became law that all deaths – whether they were suspected to be Ebola related or not – were reported to authorities and ‘medically’ buried by burial teams. Although these regulations strike at the heart of social, political, religious and economic life, by and large, the people of Sierra Leone complied.^3^ Many of the operational problems such as the lack of clean ambulances, long waits for responses to the Ebola hotline, or for burial teams, had improved. Yet much of this occurred in Guinea too.

Explanation for Sierra Leone’s turnaround lies in the synergistic relationship between national and local politics, and the way the Ebola response was able to embed public health responsibilities in locally trusted institutions. After a series of false starts, from November 2014 onwards, Sierra Leone had a comprehensive national response in place. At the centre was the National Ebola Response Centre (NERC) led by Palo Conteh, a former Minister of Defense and nephew of former president Joseph Momoh. Under Conteh the response was militarised with the Sierra Leonean Army responsible for logistics and enforcing quarantines, curfews and travel restrictions, backstopped by personnel from the British Army. The international response worked in conjunction with NERC by providing technical and operational support through a series of thematic ‘pillars’, for example on case management, burials, social mobilisation, surveillance and so on. This set up was replicated in each district of Sierra Leone in District Ebola Response Centres (DERCs). DERCs were usually headed by a combination of Sierra Leonean or British Army personnel, civil servants from the UK Department for International Development, and occasionally a Paramount Chief (PC). These arrangements considerably improved the operational aspects of the response, yet it was one which was fronted by the military and foreigners and was responsible for enforcing deeply unpopular interventions. The institutions which made this apparatus palatable were the chiefdoms.

In rural areas, chiefdoms boundaries and authorities were the key structure through which the Ebola response was mounted. Indeed for the DERCs, and the organisations working through them, chiefdom structures were an invaluable resource. Chiefdom taskforces were set up, appointed by chiefs, to carry out house to house visits, sensitisation and surveillance. These teams worked alongside district level surveillance teams. In response to the instruction to call the centralised ‘117’ hotline if someone was sick or had dead, some villagers reported that they first informed their chief who then made the decision what to do and who to call (Oosterhoff, Mokuwa, & Wilkinson, 2015). Villages, and some urban areas, imposed their own quarantines, requiring travellers and ‘strangers’ to report to chiefs. Chiefdom bylaws were imposed which banned ‘traditional’ funeral rites and home care for the sick. Initially these bylaws were instigated by chiefs in the East of Sierra Leone but the President of Sierra Leone subsequently ordered all chiefs to impose them, turning a ‘community’ response into a national strategy. Chiefs were warned that they would be fined or removed if they did not enforce the bylaws; as with smallpox their support was not assured. Bylaws were also a prominent part of the ‘action plans’ developed through the flagship social mobilisation effort, ‘Community-Led Ebola Action’, which visited 60–67% communities in Sierra Leone (Restless Development, 2015). In all these examples the arm of the central state was localised through the chieftaincy structure, which had legitimacy both among chiefdom residents and the state.

That this was possible is somewhat surprising given that the kleptocratic tendencies of chiefs, a growing issue since colonial times, is considered a key driver of the civil war, pushing a cohort of young men to revolt (Richards, 1996). Much of the violence in the war was directed at these elites. Because of these tensions, local government reform was a donor priority and post-war governments have embarked on decentralisation programmes, most notably the Local Government Act in 2004 as a means of curbing chiefly power and abuse. The Act established district councils with the responsibility for development and tax collection (Jackson, 2005). However, in most assessments the reforms have not significantly impacted on chieftaincy authority. There is now an ambiguous trilogy of politicians, chiefs and district administrators involved in local government but it is just as susceptible to elite capture and abuse (Jackson, 2007). Meanwhile villagers still voice support for a chief’s power in ‘knowing’ and securing people’s ‘rights’ (Fanthorpe, 2005).

The salient points in relation to Ebola are that recent grievances in Sierra Leone have been dominated by economic and not inter-ethnic concerns. Press reports lamented the misuse of funds: ‘All sorts of interest groups mushroomed overnight to collect cash from the Health Ministry under the guise of social mobilization’ (Kamara, 2014). On the Ministry of Health and Sanitation Facebook page comments about money were frequent. One user illustrates common suspicions well: ‘at first no cure but now people are cured stop to tell lies to this nation okay. eat what belongs to you and leave the poor (*sic*)’.^4^ Their post evokes a particularly West African idiom of consumption, and expectation of government greed. Community volunteers reported that their biggest challenge was making people believe they were not profiting from Ebola. The ‘shadow’ networks (Reno, 1995) which form the back bone of Sierra Leone’s political and economic life are assumed – and have – been lubricated with ‘Ebola money’.

Despite this evident mistrust, the suggestions of ethnically motivated plots so rife in Guinea were muted in Sierra Leone. Siaka Stevens promoted a notion of national unity (Ferme, 1998), and at the national public level there is resistance to admitting to ethnic divides (Bolten, 2014). Certainly they exist but, crucially for the response, local politics and national politics are entwined in ways which preserve ‘local’ (e.g. indigenous and ethic) rights, including not only access to land but also the practices of healing and personal protection important to cultural identity, albeit it through hierarchies which are economically exclusionary. Ebola control relied on this legitimacy. It meant that government and international operations had receptive avenues through which to intervene. Importantly, neither the interventions nor the institution (the chiefdom) were unproblematic, but they were legitimate enough to be acceptable. The building of Ebola Community Care Centres (CCCs) illustrates this tension. Located within chiefdoms, many of the 54 CCCs which were built required community donated land and labour (Oosterhoff et al., 2015). Villagers complained that they contributed work and resources to build them but were overlooked when it came to jobs, which went to those with ties to the chiefs. Yet despite these grievances over ‘elite capture’ the CCCs were still mostly well received. The model of ‘community engagement’ – through chiefdom authorities and stakeholders including the chief, speaker, elders, the women’s and youth leaders, and pervasive in development projects – was deemed acceptable and not loudly contested.

Commentators have noted the way Sierra Leoneans, especially post-war, are wise to discourses of development. Bolten (2012) argues that conflict-affected individuals in Sierra Leone do not fully accept the un-socialised ex-soldiers whom aid agencies have attempted to ‘rehabilitate’ and ‘reintegrate’ into their communities. Instead they have been ‘sensitized’ to keep their real thoughts on the matter to themselves. Acceptance of Ebola control measures, despite their continued anti-sociality, may represent a similar tendency for playing along in public, and concealing discontent beneath.

## Discussion

In Sierra Leone, colonial indirect rule and subsequent history has left the Paramount Chieftaincy, with its roots in local social orders, as the principal organising structure at the local level. In Guinea, by contrast, the legacy of French direct rule and the subsequent command economy under Sekou Toure is an administrative structure in which implementing authorities are external to the region and allied to the party in power.

When faced with an epidemic, Sierra Leone’s chiefs had sufficient legitimacy they were able to become part of the local communities of trust and play roles in the response, whereas many Guineans have been unable to trust the externally imposed civil servants who have been responsible for implementing Ebola control. Such distrust has been at its most palpable in the vicinity of mines where the external appointees (and their party) have the most to gain, and the where the inhabitants perceive the greatest exploitation and injustice of ‘their’ mineral resources.

Many caveats are necessary for such a general argument. First, one can overdraw the Sierra Leone-Guinea contrast. As hinted above, there is, perhaps, much resistance ‘latent’ in communities in Sierra Leone, where not all ‘compliance’ is compliance (just as, we have suggested, all ‘resistance’ may not be resistance). Incidents of open resistance became rare, but reports of ‘secret burials’ and of bodies being washed before burial teams were called suggests that ‘sensitization’ efforts were superficial. Quite likely, the appearance of consensus – a Mende art form (Murphy, 1990) – reflects that people are sensitised to humanitarian interventions, and the expectations and economies which accompany them. Yet this familiarity may well conceal some unresolvable tensions regarding recommendations from international organisations and elites. Another interpretation is that chiefs are not especially trusted but that the bylaw fines loomed large, reinforced by a military feared and respected in equal measure who were on hand to punish breaches.

Second, local politics can, of course, be very fractured in Sierra Leone (just as in regions of Guinea blessed in being the power-base of the regime in power, there can be harmony). At least 10 chiefs have been deposed in Port Loko (Honigsbaum, 2015). Our argument conceals the heterogeneity of chief-public relations. Some chiefs in Sierra Leone were living abroad or in Freetown, some have connections to companies and may have been visibly complicit in resource extraction. For example, the PC of Tankoro in Kono district is head of the District Ebola Response Centre, but according to Fanthorpe and Maconachie (2010) he is also on the board of local diamond mining firm Octea Mining. Such details will influence the trust ‘their people’ have in them. Neither should the cohesion of the ruling party be assumed. One of the last major incidents of violence in Sierra Leone, in Kono, revealed deep-running tensions within the ruling party, (possibly) spreading through national-local networks. In October 2014, a well-known local ‘gangster’, accused of past political violence and described as the ‘blue eyed boy’ of then Vice President Samuel Sam Sumana (who hails from Kono), incited town youth to riot to protest the transfer of his mother to an ETU (Sierra Express Media, 2015). This incident takes on particular significance because Sam Sumana was deposed in March 2015, having been campaigned against by senior Kono APC politicians, including rival Diana Konomanyi, minister of Local Government, who is in turn supported by the PC of Tankoro (Benjamin, 2015). On leaving office Sumana, whose relationship with the President had been notoriously poor, complained that he had been sidelined in the Ebola fight:

the first meeting at the break of Ebola I was not invited. I decided to text my boss [*the President]* on the 8th of July asking for invitation to involve into the Ebola planning and fight no response till now. (Jackson, 2015)

Just as with Sodality’s pre-emptive attack in Womey, instances of organised violent resistance in Sierra Leone have had political foundations.

There are also a number of other plausible explanations for the different patterns of resistance observed. One could be that the epidemic was more dramatic in Sierra Leone (and Liberia) than in Guinea. In Sierra Leone it may have been that the number of cases rose so fast, and spread across all districts, that the sheer number of deaths shocked the population into compliance. Another is that the international response was starkly divided along colonial (and quasi-colonial) lines, with France assuming responsibility for Guinea, Britain for Sierra Leone and the United States for Liberia. Perhaps there was something else qualitatively different in the styles of foreign assistance each country received through their ex-colonial power. Another is that Sierra Leone’s more recent history of international assistance meant both they and key partners already knew how to work with each other. All of these explanations may hold some weight and our argument does not exclude the possibility that they were at play too. As Cohn and Kutalek argue, ‘community resistance must be analysed in context and go beyond simple single-variable determinants’ (2016, p. 1). Our argument sheds light, however, on crucial links between local populations and national and international infrastructures, and is equally relevant to these alternative explanations.

## Conclusion

The nature and extent of social resistance to the Ebola response has contrasted strongly between Guinea and Sierra Leone. Their trajectories can be understood in relation to their contrasting political practices, namely the legacies of French direct rule and British indirect rule. This analysis – which implicates the patronage networks between central and local state actors – may explain why Sierra Leone has seen less overt resistance and why much of the rumour in Sierra Leone has been about money-making not ethnically motivated genocide which has been a preoccupation in Guinea. For both Guinea and Sierra Leone it demonstrates some specific ways in which often noted politics play into control measures, and elude ‘public’ and ‘community’ engagement efforts. That distinctions existed between the ‘publics’ of public health and the constituents of local communities of trust is not surprising, but of significance is the way that authorities in each country were more or less able to forge links between them. The implications for epidemic response more broadly are that an understanding of specific historical styles of state administration and, relatedly, the symbolism of the messenger will contribute to identifying trusted local actors and institutions and working in ways which do not exacerbate antagonism. In no setting can leaders or faultlines be assumed. Instead historically and anthropologically informed enquiries, which proved successful even in the most troubled parts of Guinea (see Anoko, 2014), need to be conducted to identify salient local politics before intervening, and with interventions proceeding with these sensitivities in mind.

## Disclosure statement

No potential conflict of interest was reported by the authors.

## Funding

This work was supported by the UK Department for International Development [Future Health Systems: Delivering Effective Health Services Research Programme Consortia Contract Number PO-5467]; Wellcome Trust/UK Department for International Development ‘Research for Health in Humanitarian Crises’ (R2HC) [PHGHZE82 10].

## Notes

1. WHO Ebola Situation Reports are at: http://apps.who.int/ebola/ebola-situation-reports.

2. For more details see http://www.ebola-anthropology.net/.

3. There are reports of corpses being washed and people escaping quarantine but persistent extreme and open opposition is rare. Situation reports from Sierra Leone can be found at: http://www.nerc.sl/?q=situation-report.

4. Posted by ‘Mohamed Bundu’ on Sierra Leone’s Ministry of Health and Sanitation’s Facebook page. 28 July 2014, at 05.45 am.
